# Anthropometric Indicators of Body Composition Associated With Lipid and Glycemic Profiles in Overweight Brazilian Children and Adolescents From 2008 to 2020

**DOI:** 10.3389/fnut.2022.908562

**Published:** 2022-06-09

**Authors:** Carlos Alberto Nogueira-de-Almeida, Fábio da Veiga Ued, Andrea Aparecida Contini, Edson Zangiacomi Martinez, Luiz Antonio Del Ciampo, Maria Eduarda Nogueira-de-Almeida, Ivan Savioli Ferraz, Raquel Farias Barreto Silva, Elza Daniel de Mello, Mauro Fisberg

**Affiliations:** ^1^Medical Department, Federal University of São Carlos, São Carlos, Brazil; ^2^Nutrition Department, Faculty of Medicine of Ribeirão Preto, University of São Paulo, São Paulo, Brazil; ^3^Faculty of Medicine of Ribeirão Preto, University of São Paulo, São Paulo, Brazil; ^4^Pediatric Department, Faculty of Medicine of Ribeirão Preto, University of São Paulo, São Paulo, Brazil; ^5^Pediatrics Department, Federal University of Rio Grande Do Sul, Porto Alegre, Brazil; ^6^Pensi Institute, Hospital Infantil Sabará, José Luiz Egídio Setúbal Foundation, São Paulo, Brazil; ^7^Pediatrics Department, Federal University of São Paulo, São Paulo, Brazil

**Keywords:** obesity, anthropometry, body composition, nutritional status, lipid profile, glycemic profile

## Abstract

**Background:**

Anthropometric indicators have been used to predict health problems. The objective was to determine which indicators present better correlation with dyslipidemia, hyperglycemia and peripheral insulin resistance, as well as the cutoff points capable of predicting lipid and glycemic alterations in Brazilian children and adolescents.

**Methods:**

A cross-sectional study conducted with 568 overweight individuals, aged between 5 and 18 years, living in Southeast and South Brazilian regions, submitted to anthropometric and body composition evaluation by bioimpedance, in addition to fasting laboratory tests [total cholesterol (TC), triglycerides (TG), low-density lipoprotein cholesterol (LDL-c), high-density lipoprotein cholesterol (HDL-c), fasting glycemia, and homeostasis model assessment–insulin resistance (HOMA-IR)]. Pearson's correlation was used to evaluate the association between anthropometric indicators and serum biomarkers. The ROC curve with Youden's J index was used to suggest anthropometric cutoff points with better ability to predict or rule out lipid and glycemic changes.

**Results:**

Cutoff points obtained for the z-score of body mass index (BMI), waist circumference (WC), and waist circumference for height (WC/H) showed high specificity (52 to 87%) and low sensitivity (23 to 59%), indicating greater ability to exclude changes in HDL-c, TG, and HOMA-IR levels. Cutoff points suggested for BMI ranged from +1.86 to +2.20 z-score. WC cutoff points ranged from +1.29 to +1.72, and, for the WC/H index, from +1.21 to +1.25. It was suggested the use of the following cutoff points to rule out changes in HDL-c, TG, and HOMA-IR values in clinical practice: BMI < z-score +2 and WC/H < z-score +1.29. In body fat percentage (BFP) analyses, the cutoff point < of 34% may be able to rule out changes in HDL-c (specificity of 70%), while the cutoff point > 36.6% may be able to predict changes in the HOMA-IR index (sensitivity of 76%).

**Conclusion:**

It is not yet possible to state which anthropometric parameter has the best correlation with lipid and glycemic alterations in overweight children and adolescents. We suggest considering BMI, WC, and WC/H cutoff points together to rule out changes in HDL-c, TG, and HOMA-IR, and use the BFP cutoff point to predict changes in HOMA-IR.

## Introduction

Obesity in childhood and adolescence is associated with several comorbidities, including dyslipidemia, peripheral insulin resistance, non-alcoholic fatty liver disease, polycystic ovary syndrome, psychosocial disorders, dermatitis, orthopedic problems, arterial hypertension, among others (1). Anthropometric and body composition parameters used for the diagnosis of obesity, such as waist circumference (WC) and body fat percentage (BFP), and indices, such as body mass index (BMI) and waist circumference for height (WC/H), have also been used to predict the coexistence of cardiometabolic risk factors (2–4). However, there is still no widely accepted definition of which of them is the best indicator, with the aggravating factor that different cut-off points have been used for each of them.

BMI can be assessed using reference curves published by the World Health Organization (WHO) and correlates with glucose and lipid profiles (5). For children over 5 years of age, BMI z-score values between +1 and +2 are used as the definition of overweight and obesity when >+2 (6). The data obtained in the WC measurement and WC/H index can be compared with age- and gender-specific cut-off points, using reference values defined by the National Health and Nutrition Examination Survey (NHANES) III (4), and values above z-score +2 can be considered high (4). Some authors, especially when WC is used as one of the components of the metabolic syndrome (MS), suggest the 90th percentile as a cut-off point for values that indicate the presence or absence of excess weight in an individual (7), which is approximately equivalent to the z-score of +1.29 (8), and this idea can also be expanded to the WC/H index.

In view of the above, the present study aims to determine which anthropometric indicator (BMI, WC, WC/H, and BFP) presents the best correlation with dyslipidemia, hyperglycemia, and peripheral insulin resistance, as well as the ideal cutoff point for anthropometric parameters capable of predicting or ruling out lipid and glycemic alterations in Brazilian children and adolescents.

## Methods

### Study Design and Places

This is all cross-sectional study with data obtained from electronic medical records of patients from two health services located in two different regions of Brazil and with different socioeconomic profiles. Site 1: a clinic specialized in nutritional diseases and serving clients with health insurance in the southeast region of the country in the municipality of Ribeirão Preto/São Paulo. Site 2: patients from the public health system of the reference clinic in obesity in a university hospital in the southern region of the country in the municipality of Porto Alegre/Rio Grande do Sul. This study was approved by the Research Ethics Committee of the Federal University of São Carlos (Nos. 4,133,407).

### Study Population

Data collected at the first consultation during the years 2008 to 2020 and patients aged between 5 and 18 years and BMI above the z+1 score (*n* = 1,296 eligible) were included. Sampling was not performed because all the patients seen at the first visit in the time interval were initially eligible. Exclusion criteria were: presenting other diseases, such as type 1 diabetes mellitus (*n* = 1), hypothyroidism (*n* = 6), and inborn errors of metabolism (*n* = 1); unmeasured abdominal measure (*n* = 89); non-performing bioimpedance test (*n* = 290); and laboratory tests not available (*n* = 341). In the following criteria, 568 individuals participated in the study, 326 of which were attended in Locals 1 and 242 in Local 2. Therefore, a convenience sample was adopted.

### Data Collection and Analysis

Anthropometric evaluation followed a standardized technique. Weight and height measurements followed WHO recommendations (6). All the patients underwent anthropometric and body composition evaluation by bioimpedance at the first visit, at which time they were asked to collect laboratory tests with 12 h of fasting, and an interval of up to 30 days was tolerated for return with the requested tests. The bioimpedance test was performed on Biodynamics® 310 equipment in both sites, and it was adequately validated (9).

BMI cutoff points for defining excessive adiposity were: (a) z-score > +1 and ≤ +2, and (b) z-score > +2 (6). The WC cutoff points and the WC/H index for defining excessive adiposity were: a) z-score ≥ +1.29 and ≤ +2 (equivalent to 90 and 97th percentiles), and b) z-score > +2 (4). BFP was considered elevated when it was above the 85th percentile (10).

The laboratory tests of Site 1 were performed in one of the three laboratories in the city that were used under the free choice of the family, and all the laboratories constituted certified institutions that used similar methods and kits. The laboratory tests of Site 2 were performed in the laboratory of the university hospital. The serum biomarkers measured were fasting glucose, fasting insulin, total cholesterol (TC), low-density lipoprotein cholesterol (LDL-c), high-density lipoprotein cholesterol (HDL-c), and triglycerides (TG). The Homeotasis Model Assessment-Insulin Resistance (HOMA-IR) index was determined. Two tubes of 4 ml of blood were collected in a vial without additive and sent within 2 h to the laboratory for the processing of samples and biochemical and hormonal analyses. The biological material was separated into a Bio Eng® centrifuge model BE 4,000 for 5 min at 3,500 rpm between one and 2 and 1/2 h after collection (sufficient time for blood coagulation). The biochemical dosage of insulin was performed in one of the aliquots on the same day of the collection by the chemiluminescence method, with automation by the Immulitte DPC Medlab equipment®. Glycemia and lipidogram were evaluated by the enzymatic method with Cobas Mira Plus Roche automation equipment®.

Hyperglycemia was considered when plasma glucose value was above 99 mg/dL and insulin resistance when HOMA-IR corrected for age and sex was above the cutoff points previously defined among Brazilian children and adolescents: 5 to 8.9 years: ≥1.76 (boys) and ≥1.39 (girls); 9 to 10.9 years: ≥1.97 (boys) and ≥2.62 (girls); 11 to 12.9 years: ≥2.65 (boys) and ≥3.02 (girls); 13 to 14.9 years: ≥3.21 (boys) and ≥3.46 (girls); 15 to 17.9 years: ≥2.39 (boys) and ≥2.89 (girls) (11, 12). The cutoff points for defining dyslipidemia were: CT > 170 mg/dl; LDL-c >110 mg/dl; HDL-c <45 mg/dl; and TG > 75 mg/dl (1-9 years) or >90 (10 to 18 years) (13).

### Statistical Analysis

The prevalence of individuals with elevated serum biomarkers was compared according to the cut-off points of different criteria for defining excessive adiposity using the Pearson chi-squared test. Pearson correlation coefficients (r) with corresponding 95% confidence intervals (95% CI) were used to assess the association between anthropometric indicators of excessive adiposity and serum biomarkers. The linearity assumption was visually checked by scater plots of these variables.

Analyzes of the Receiver Operating Characteristic (ROC) curve with Youden's J index (14) were used to suggest the cut-off point for the z-score of BMI, WC and WC/H, and for the BFP, with greater ability to predict or rule out changes in lipids and blood glucose. The Youden index is defined as J = max_c_ [sensitivity (c) + specificity (c) – 1] for given cut-off Point c in an ROC curve corresponds to the maximum distance from the diagonal line from (0, 0) to (1, 1). The ROC curves and outcome analyses were performed using the pROC package of R statistical software.

## Results

### Population Characteristics

A total of 568 individuals were evaluated, 50.5% (*n* = 287) were female, and the mean age was 11.7 years (standard deviation, 2.7 years; range, 5.2 to 17.9 years). It was observed that 63.7% (*n* = 362) of the individuals were classified as obese (BMI > z score, +2). In the analysis of the prevalence of glycemic alterations, 63.7% (*n* = 362) of the individuals had high HOMA-IR values against only 9.2% (*n* = 52), with high fasting glucose. As for the lipid profile, the biomarker with the highest prevalence of serum inadequacy was TG (56%, *n* = 318).

### Correlation Between Anthropometric Indicators and Lipid and Glycemic Changes

The raw values of the z-score of the anthropometric indicators BMI, WC, and WC/H showed a weak correlation with the serum levels of HDL-c, TG, and with the values of HOMA-IR. BFP was also correlated with serum HDL-c levels and with HOMA-IR values, showing a moderate correlation (*r* = 0.408, *p* < 0.001) (Table 1).

**Table 1:** Correlation (r) between anthropometric indicators of excessive adiposity and serum biomarkers.

	**TC r (*p*-value) (95%CI)**	**LDL-c** **r (*p*-value)** **(95%CI)**	**HDL-c r (*p*-value) (95%CI)**	**TG** **r (*p*-value)** **(95%CI)**	**Blood glucose r (*p*-value) (95%CI)**	**HOMA-IR** **r (*p*-value)** **(95%CI)**
BMI (z-score)	-0.041 (-0.123, 0.042)	−0.034 (-0.116, 0.049)	-0.159* (-0.238,−0.078)	0.108*** (0.026, 0.188)	-0.047 (-0.129, 0.035)	0.221* (0.141, 0.298)
WC (z-score)	0.030 (-0.053, 0.112)	0.015 (-0.067, 0.097)	-0.121** (-0.201,−0.039)	0.163* (0.082, 0.242)	0.034 (-0.048, 0.116)	0.131** (0.049, 0.211)
WC/H (z-score)	0.037 (-0.046 , 0.118)	0.031 (-0.052 , 0.113)	-0.147* (-0.226 , -0.065)	0.151* (0.069 , 0.230)	0.013 (-0.070 , 0.095)	0.128** (0.046 , 0.208)
BFP (percentile)	-0.067 (-0.149, 0.015)	−0.009 (-0.091, 0.073)	-0.165* (-0.244,−0.084)	0.028 (-0.054, 0.110)	0.006 (-0.077, 0.088)	0.408* (0.338, 0.475)

^*^p < 0.001. ^**^p < 0.005. ^***^p < 0.05.

In addition, the prevalence of individuals with dyslipidemia, high blood glucose, and insulin resistance was analyzed according to different cutoff points already established in the scientific literature for BMI, WC, WC/H, and BFP (Table 2). The BMI analysis showed that the z-score > +2 cut-off point was able to identify a greater proportion of individuals with alterations in HDL-c and HOMA-IR, compared to the z-score cut-off > +1 and ≤ +2. Analysis of WC and WC/H index showed that both cut-off points, z-score ≥ +1.29 and z-score > +2, were able to identify a greater number of obese children with low HDL-c and elevated TG and HOMA-IR, compared to those with no alterations in these anthropometric parameters. On the other hand, values situated between the cut-off points z-score ≥ +1.29 and ≤ +2 for WC and WC/H were more suitable for identifying individuals with high fasting glucose. The BFP above the 85th percentile identified a higher percentage of individuals with alterations in HOMA-IR, compared to individuals classified below this percentile (Table 2).

**Table 2:** Prevalence of dyslipidemia, hyperglycemia, and insulin resistance according to the cutoff points of different criteria for defining excessive adiposity.

	***n***	**high TC**	**high LDL-c**	**lowHDL-c**	**high TG**	**Elevatedfastingblood glucose**	**high HOMA-IR**
		***n* (%)**	***n* (%)**	***n* (%)**	***n* (%)**	***n* (%)**	***n* (%)**
Total population	568	229 (40.3)	192 (33.8)	316 (55.6)	318 (56.0)	52 (9.2)	362 (63.7)
**Anthropometric indicators and cut-off points**							
BMI (+1 < z score ≤ +2)	206	79 (38.3)	68 (33.0)	96 (46.6)	107 (51.9)	21 (10.2)	99 (48.1)
BMI (z score > +2)	362	150 (41.4)	123 (34.0)	220 (60.8)	211 (58.3)	31 (8.6)	263 (72.7)
P-value, chi-squared test		0.47	0.81	<0.01	0.14	0.52	<0.01
WC (z score > +2)	149	62 (41.6)	49 (32.9)	88 (59.1)	98 (65.8)	9 (6.0)	106 (71.1)
WC (+1.29 ≤ z score ≤ +2)	323	136 (42.1)	113 (35.0)	189 (58.5)	181 (56.0)	39 (12.1)	212 (65.6)
WC (z score < +1.29)	96	31 (32.3)	29 (30.2)	39 (40.6)	39 (40.6)	4 (4.2)	44 (45.8)
*P*-value, chi-squared test		0.21	0.67	<0.01	<0.01	0.02	<0.01
WC/H (z score > +2)	121	51 (42.1)	39 (32.2)	68 (56.2)	82 (67.8)	7 (5.8)	86 (71.0)
WC/H (+1.29 ≤ z score ≤ +2)	319	135 (42.3)	114 (35.7)	193 (60.5)	180 (56.4)	41 (12.9)	216 (67.7)
WC/H (z score < +1.29)	128	43 (33.6)	38 (29.7)	55 (43.0)	56 (43.8)	4 (3.1)	60 (46.9)
*P*-value, chi-squared test		0.21	0.44	<0.01	<0.01	<0.01	<0.01
BFP (percentile> 85)	538	218 (40.5)	178 (33.1)	301 (55.9)	303 (56.3)	51 (9.5)	352 (65.4)
BFP (percentile <85)	30	11 (36.7)	13 (43.3)	15 (50.0)	15 (50.0)	1 (3.3)	10 (33.3)
*P*-value, chi-squared test		0.68	0.25	0.52	0.50	0.26	<0.01

### Cut-Off Points for Anthropometric Indicators Capable of Predicting or Ruling Out Lipid and Glycemic Changes

**Figure 1:**
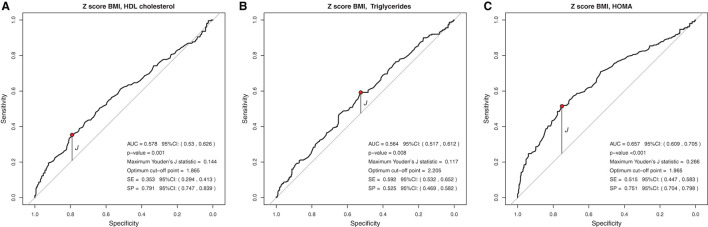
**(A–C)** Suggested cutoff points for the BMI z-score, with greater ability to rule out changes in HDL-c, TG, and HOMA-IR levels.

**Figure 2:**
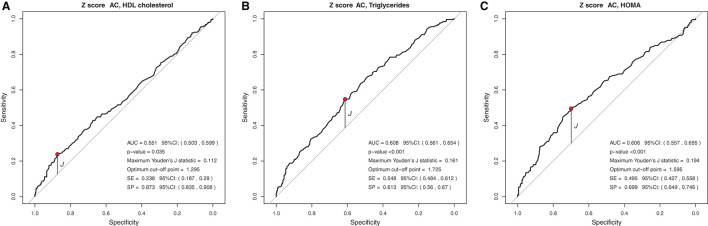
**(A–C)** Suggested cut-off points for a WC z-score, with greater ability to rule out changes in HDL-c, TG, and HOMA-IR levels.

**Figure 3:**
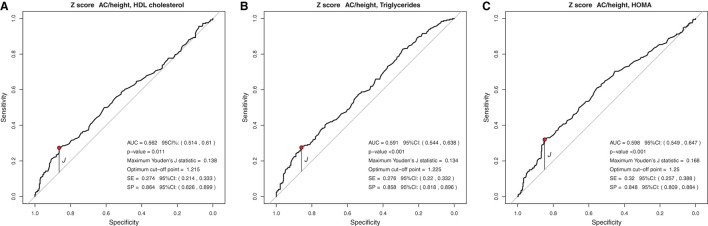
**(A–C)** Suggested cut-off points for the WC/H z-score, with greater ability to rule out changes in HDL-c, TG, and HOMA-IR levels.

In the analysis of the ROC curve, the cutoff points obtained by the Youden J index for the z-score of BMI, WC, and WC/H showed high specificity (52 to 87%) and low sensitivity (23 to 59%), indicating that individuals without lipid and glycemic alterations have a high probability of being below the cut-off point found. The cutoff points suggested for the BMI in the population studied, capable of ruling out changes in HDL-c, TG, and HOMA-IR levels, were z-score < +1.86, z-score < +2.20, and z-score < +1.96, respectively (Figure 1). The cutoff points suggested for WC, capable of ruling out changes in HDL-c, TG, and HOMA-IR levels, were z-score < +1.29, z-score < +1.72, and z-score < +1, 59, respectively (Figure 2). And the cutoff points suggested for the WC/H index, capable of ruling out changes in HDL-c, TG, and HOMA-IR levels, were z-score < +1.21, z-score < +1.22, and z-score < +1.25, respectively (Figure 3). These cut-off points are also shown in Table 3.

**Table 3:** Cutoff points according to Younden's “J” index analysis to rule out or predict biochemical abnormalities or body fat percentage obtained from data from children and adolescents aged 5 to 18 years from two Brazilian overweight and obesity outpatient clinics.

**Indicator**	**Biomarker**	**Cutoff point**
Body mass index	HDL-c	< +1.86 z-score
	Triglycerides	< +2.20 z-score
	HOMA-IR	< +1.96 z-score
Waist circumference	HDL-c	< +1.29 z-score
	Triglycerides	< +1.72 z-score
	HOMA-IR	< +1.59 z-score
Waist circumference/Height	HDL-c	< +1,21 z-score
	Triglycerides	< +1.22 z-score
	HOMA-IR	< +1.25 z-score
Body fat percentage	HDL-c	<34.0%
	HOMA-IR	>36.6%

*HDL-c, high-density lipoprotein cholesterol; HOMA-IR, homeostatic model assessment of insulin resistance*.

**Figure 4:**
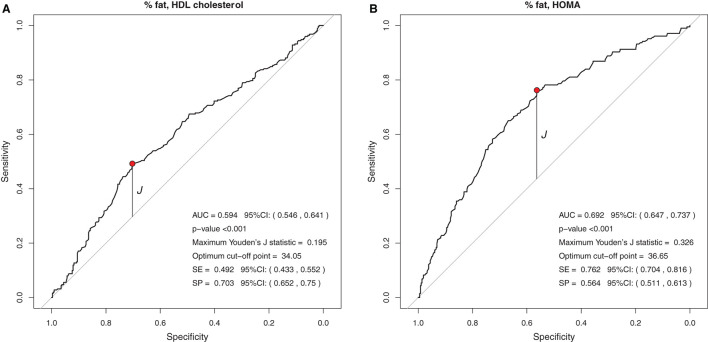
**(A,B)** Suggested cutoff points for BFP, with greater ability to rule out changes in HDL-c levels and to predict changes in HOMA-IR.

Regarding the BFP, the cut-off point obtained by the Youden J index showed low specificity and high sensitivity for HOMA-IR, indicating that individuals with insulin resistance have a high probability of being above the cut-off point. The suggested cutoff point for the BFP with the greatest ability to predict elevated HOMA-IR in Brazilian children and adolescents was 36.6% (Figure 4).

## Discussion

Obesity is a chronic nutrineurometabolic disease related to cardiometabolic events, (15) which may not be evident in some patients (16). Therefore, it is important to use anthropometric parameters as a low-cost tool, viable in clinical practice and of simple interpretation for cardiometabolic risk assessment in obese pediatric patients. BMI has been used by professionals worldwide as the main indicator of the presence of obesity and its comorbidities. Although it depends on two measures and a mathematical calculation, its use has become increasingly embedded in pediatric practice worldwide, especially since the publication of the WHO growth charts (6). Several countries adopt BMI curves in official childcare instruments, and professionals are accustomed to their use and interpretation. The WC, despite the extreme ease of measurement, still depends on the use of curves or tables that are not widespread. The same can be said of the WC/H index, which still depends on the additional measurement of height. On the other hand, BFP, which should be the gold standard, considering that obesity is about excess adiposity, has its measurement even more complex, because it depends on equipment rarely presented in pediatric clinics and also depends on little known curves or tables. The present study aimed to identify, among the anthropometric and body composition parameters used in clinical practice and useful in the definition of obesity, the one that best correlates with alteration in biomarkers for dyslipidemia, hyperglycemia, and peripheral insulin resistance.

The z-score values for BMI, WC, and WC/H showed significant correlation with three serum biomarkers (HDL-c, TG, and HOMA-IR), while BFP correlated with two biomarkers (HDL-c and HOMA-IR). However, except for BFP, which showed moderate correlation with HOMA-IR, all other correlations were weak. Similar results were found by Faria et al. (17) in Brazilian adolescents and by Vásquez et al. (18) in a cohort of Chilean children aged 4, 7, and 10 years; however, in both studies, not all the participants were overweight. The observation of a moderate correlation between body fat and HOMA-IR was also observed in a study of overweight Saudi Arabian children and adolescents aged between 2 and 20 years (19). Excess body fat can lead to metabolic changes, including inflammatory mechanisms, which increases insulin resistance and may help to partially explain this finding.

A positive association between BMI and TG was also observed by Ejtahed et al. (20) among Iranian adolescents. In China, in a study carried out with 2,243 overweight individuals aged between 7 and 17 years, it was observed that BMI was the best predictor of dyslipidemia in the group studied (21). In Brazil, it was observed that BMI was a significant predictor of dyslipidemia in a study with 874 children and adolescents aged 6 to 19 years in Belém (PA) (22). In our study, it was not possible to state that BMI is the best predictor of dyslipidemia and insulin resistance, suggesting that WC measurements and BFP analysis should also be considered in routine pediatric screening.

It is known that WC measurements are an effective indicator of central fat distribution, serving as a strong marker of cardiometabolic risk in the pediatric population (23, 24). In a multicenter study by da Silva et al. (25) in 2018, with 520 adolescents, it was also observed a positive association between WC and HOMA-IR. The study by Khoury et al. (26), carried out with more than 14,000 North American children and adolescents aged 5 to 18 years, also showed a positive association between the WC/H index and HDL-c and HOMA-IR levels. The authors also highlighted that individuals with high WC/H and BMI had higher cardiovascular risk compared to those with only high BMI and normal WC/H, confirming the importance of WC measurement in clinical practice.

Faria et al. (17) highlighted that BMI, WC, and WC/H were good predictors of excess body fat. Aristizabal et al. (27) found a moderate correlation of BMI, WC, and WC/H with HOMA-IR in Colombian children aged between 2 and 5 years. These data show the need to use several anthropometric parameters together for decision-making in clinical practice.

The attempt to find anthropometric indices of adiposity that can predict metabolic risk changes for the development of cardiovascular diseases has produced heterogeneous results. We observed that no anthropometric indicator was associated with serum levels of TC, LDL-c, and fasting blood glucose. It should be noted that the CT and LDL-c tests do not compose the MS picture. These findings are different from those found by Oliosa et al. (3), who found a positive association between WC/H and BFP measurements with high CT values.

Regarding fasting blood glucose, this is an exam used in the diagnosis of MS, but this study did not find an association with the anthropometric indicators studied, as well as the study by de Quadros et al. (2). The glycemic alteration is unusual in obese Brazilian children, perhaps due to the so-called “metabolically healthy obese,” (28) which presents some normal exams and lower prevalence of changes in fasting blood glucose.

Our analyses of the prevalence of lipid and glycemic alterations according to anthropometric cutoffs already established in the literature positively evidenced the use of the z-score + 2 cutoff points for BMI and the 85th percentile for BFP in the detection of individuals with metabolic alterations. For WC and WC/H parameters, the cutoff z-score +1.29 was able to identify a greater proportion of individuals with biochemical alterations. Other studies have also used the same cutoff point for BMI (27) and WC (26, 29). As for the WC/H index, the authors still preferred to use the cutoff point of 0.5 (26, 30).

In the analysis of the ROC curves and Youden's J index, this study observed that the suggested cutoff points for the BMI varied between +1.86 and +2.20 z-scores, close to the cutoff point (z-score +2) already used by WHO for diagnosis of obesity. WC cutoff points ranged between +1.29 and +1.72, while, for the WC/H index, they ranged between +1.21 and +1.25, all also close to the z-score +1.29, used by some authors for predicting excess body fat. Therefore, for clinical practice, we suggest using the following cutoff points to rule out changes in HDL-c, TG, and HOMA-IR values: BMI < z-score +2 and WC and WC/H < z-score +1.29.

In BFP analyses, the cutoff point <34% of body fat may be able to rule out changes in HDL-c (high specificity), while the cutoff point > 36.6% may be able to predict changes in the HOMA-IR index (high sensitivity). In the study by Abdelhamed et al. (19), a cut-off of 46.1% of BFP was shown to have the best sensitivity/specificity ratio for detecting obesity-related morbidities in Saudi children and adolescents. The joint analysis of several biochemical parameters (grouped as “obesity-related morbidities”) to estimate the accuracy of the BFP in predicting laboratory changes in overweight individuals may have contributed to the difference between the Saudi work and the present study.

It should be noted that, in an attempt to establish new anthropometric cutoffs capable of predicting or ruling out dyslipidemia and glycemic alterations, the interpretation of the area under the curve (AUC) value should be considered (31). While studying overweight individuals aged between 2 and 20 years in Saudi Arabia, Abdelhamed et al. (19) found AUC between 0.638 and 0.657 for the parameters BFP, WC, and BMI capable of predicting “obesity-related morbidities,” a condition that included, in addition to dyslipidemia, pre/hypertension, pre/diabetes, and hypovitaminosis D. In a study involving 3,327 overweight European children and adolescents aged 3 to 16 years, the authors found an AUC of 0.57, 0.58, and 0.59 when analyzing the BFP values capable of predicting changes in HDL-c, LDL-c, and TG, respectively; in this study, the authors found no differences between the AUCs of BFP and BMI (32).

Similar to the findings above, we observed in our study that BMI, WC, and WC/H had similar AUC values between 0.551 and 0.657, but with high specificity and low sensitivity, evidencing the ability to rule out changes in HDL-c, TG, and HOMA-IR. Thus, it seems that, when considered together, the cutoff points suggested for BMI, WC, and WC/H are capable of excluding, with moderate accuracy, alterations in the lipid profile and insulin resistance of Brazilian children and adolescents with excess of weight. If it is not possible to use these anthropometric indicators together in clinical practice, it is possible to use at least one of them (for example, BMI) in view of the similar ability of all of them to correlate with biochemical changes. As for the BFP, the AUC was 0.594 and 0.692 for HDL-c and HOMA-IR, respectively. The analyses between BFP and HOMA-IR showed high sensitivity, indicating a greater ability of the suggested cutoff point for BFP (>36.6%) to predict changes in HOMA-IR.

Among the strengths of the current study, the heterogeneity of our population stands out, which comprised individuals from a private clinic, which mainly serves middle- and upper-class populations, and from a public outpatient clinic, which serves low-income populations, in two different regions of Brazil. As for the limitations, it is noteworthy that the individuals studied came from reference services in the follow-up of children and adolescents with obesity, and these patients tend to have more severe disease, a fact that may influence the results. In addition, the level of physical activity was not analyzed. Complex metabolic interactions and other variables (e.g., the level of physical activity) that interfere with lipoprotein metabolism may help to explain the absence of a strong correlation between anthropometric parameters and the lipid profile in the current study. Finally, a dietary intake assessment would, perhaps, affect the data analysis as a cofactor, but our data set did not include this evaluation.

## Conclusion

The z-score values for BMI, WC, and WC/H correlated with HDL-c, TG, and HOMA-IR, while BFP correlated with HDL-c and HOMA-IR. It is still not possible to say which anthropometric parameter has the best correlation with lipid and glycemic changes in obese Brazilian children and adolescents. The cutoff points for BMI, WC, and WC/H capable of ruling out alterations in HDL-c, TG, and HOMA-IR found in the present study were close to those already classically used in the scientific literature to define the indicator as altered or not. We suggest considering the BMI, WC, and WC/H cut-off points together to rule out changes in HDL-c, TG, and HOMA-IR, and using the BFP cut-off point to predict changes in HOMA-IR.

## Data Availability Statement

The raw data supporting the conclusions of this article will be made available by the authors, without undue reservation.

## Ethics Statement

The studies involving human participants were reviewed and approved by Research Ethics Committee of the Federal University of São Carlos (number 4,133,407). Written informed consent for participation was not provided by the participants' legal guardians/next of kin because: We used data base information with no patient identification.

## Author Contributions

CN-d-A: conception, design, acquisition, analysis, interpretation, drafting, final approval of the version to be published, figures, study design, data collection, data interpretation, and data analyses. MF and AC: final approval of the version to be published, literature search, data analysis, and data interpretation. EDM: revising, final approval of the version to be published, literature search, data analysis, and data interpretation. RS: acquisition, analysis, or interpretation of data for the work, literature search, drafting, final approval of the version to be published, and data collection. IF: final approval of the version to be published, literature search, and drafting. MN-d-A: design of the work or the acquisition, analysis, drafting, and final approval. LD: conception or design of the work, drafting, final approval of the version to be published, data interpretation, and data analyses. EZM: analysis, or interpretation of data for the work, drafting, final approval of the version to be published, data interpretation, data analysis, and figures. FU: conception, interpretation, drafting, and final approval of the version to be published data interpretation. All authors contributed to the article and approved the submitted version.

## Conflict of Interest

The authors declare that the research was conducted in the absence of any commercial or financial relationships that could be construed as a potential conflict of interest.

## Publisher's Note

All claims expressed in this article are solely those of the authors and do not necessarily represent those of their affiliated organizations, or those of the publisher, the editors and the reviewers. Any product that may be evaluated in this article, or claim that may be made by its manufacturer, is not guaranteed or endorsed by the publisher.
